# Living on the edge: Crayfish as drivers to anoxification of their own shelter microenvironment

**DOI:** 10.1371/journal.pone.0287888

**Published:** 2024-01-02

**Authors:** Adrian Neculae, Zanethia C. Barnett, Kristian Miok, Marcelo M. Dalosto, Iryna Kuklina, Tadashi Kawai, Sandro Santos, James M. Furse, Ovidiu I. Sîrbu, James A. Stoeckel, Lucian Pârvulescu

**Affiliations:** 1 Faculty of Physics, West University of Timisoara, Timisoara, Romania; 2 Center for Bottomland Hardwoods Research, Southern Research Station, USDA Forest Service, Oxford, MS, United States of Ameirca; 3 Crayfish Research Centre, Institute for Advanced Environmental Research, West University of Timisoara, Timisoara, Romania; 4 Laboratório de Carcinologia, Programa de Pós-Graduação em Biodiversidade Animal, Universidade Federal de Santa Maria, Santa Maria, RS, Brazil; 5 South Bohemian Research Center of Aquaculture and Biodiversity of Hydrocenoses, Faculty of Fisheries and Protection of Waters, University of South Bohemia in České Budějovice, Vodňany, Czech Republic; 6 Central Fisheries Research Institute, Yoichi, Hokkaido, Japan; 7 Coastal and Marine Research Centre, Griffith University, Gold Coast, Queensland, Australia; 8 Department of Biochemistry and Pharmacology, Faculty of Medicine, “Victor Babeș” University of Medicine and Pharmacy Timisoara, Timisoara, Romania; 9 School of Fisheries, Aquaculture, and Aquatic Sciences, Auburn University, Auburn, AL, United States of America; 10 Department of Biology-Chemistry, Faculty of Chemistry, Biology, Geography, West University of Timisoara, Timisoara, Romania; VIT University, INDIA

## Abstract

Burrowing is a common trait among crayfish thought to help species deal with adverse environmental challenges. However, little is known about the microhabitat ecology of crayfish taxa in relation to their burrows. To fill this knowledge gap, we assessed the availability of oxygen inside the crayfish shelter by series of *in-vivo* and *in-silico* modelling experiments. Under modeled condition, we found that, except for the entrance region of the 200 mm, a flooded burrow microenvironment became anoxic within 8 h, on average. Multiple 12-hour day-night cycles, with burrows occupied by crayfish for 12 h and empty for 12 h, were not sufficient for refreshing the burrow microenvironment. We then examined the degree to which crayfish species with different propensities for burrowing are tolerant of self-created anoxia. From these experiments, primary and secondary burrowers showed best and most consistent tolerance—exhibiting ≥ 64% survival to anoxia and 25–91% survival of ≥ 9 h at anoxia, respectively. Tertiary burrowers exhibited little to no tolerance of anoxia with 0–50% survival to anoxia and only one species exhibiting survival (2%) of ≥ 9 h at anoxia. Results suggest that moderate to strongly burrowing crayfish can quickly draw down the dissolved oxygen in burrow water but appear to have conserved a legacy of strong tolerance of anoxia from their monophyletic ancestors–the lobsters–whereas tertiary burrowers have lost (or never evolved) this ability.

## Introduction

Sheltering is used by numerous animals for protection of themselves and offspring against various environmental stressors or biological competitors [1, 2]. Whether they exploit existing refuges or construct them de novo, the animals invest energy not only to build, but to maintain and defend their refuges [3, 4]. A shelter’s microenvironment might play an evolutionary ecological role in the history of a species [5–7], but more research is needed to understand the potential feedbacks between constructed environments and evolution [8, 9]. Although crayfish are among the largest freshwater invertebrates, they are vulnerable to predators and many species require the use of different kinds of burrows as shelters [7]. In-depth research addressing the microenvironment of crayfish burrows may uncover important ecological, behavioral, and, to some extent, even evolutionary relationships.

Evolution should not be ignored when conserving a species [10, 11]. Both ecological and evolutionary aspects are essential [12]. Burrowing behavior and ecology differ widely among the over 540 documented, geographically dispersed species of crayfish [13, 14]. There is currently no evidence supporting phylogenetic divergences between the different ecological types of burrowing crayfish [13, 14]. Based on their degree of dependence on burrows, crayfish can be categorized as primary, secondary, and tertiary burrowers [15, 16]. Primary burrowers live in swamps and marshes or hydrated soils of floodplains and prairies, and spend almost their entire lives in complex, deep burrows, often with no connection to open water and the groundwater/air interface frequently occurring deep within the burrow. Secondary burrowers periodically shift between surface water and burrow habitats. Burrows are less complex and frequently exhibit lateral connections to nearby still or running surface waters (lakes, ponds, streams, rivers) with burrows more strongly flooded than primary burrows or even completely flooded in the case of lateral burrows in the stream banks. Tertiary burrowers spend most of their lives in surface waters but are capable of digging simple burrows in response to dewatering during environmental extremes [17]. These categories represent a gradient, rather than clear breakpoints, in burrowing behavior with different populations of the same species sometimes reported as belonging to more than one burrowing category (i.e., primary/secondary burrower, secondary/tertiary burrower).

Hypoxia is one of the common problems in aquatic environments [18, 19] that can have major impacts on populations of some crayfish species due to their high oxygen demands [20–22]. Other crayfish species have been documented as withstanding oxygen depletion for long periods [23, 24], and empiric observations suggest that even species that are considered sensitive to hypoxia may persist in poorly oxygenated waters [25].

Water in crayfish burrows was found ranging from hypoxic to near anoxic [26]. It is generally believed that in-burrow water oxygen saturation depends on crayfish activity, as well as on burrow structure [27, 28] and habitat stability [29, 30]. Relationships between crayfish burrowing behavior and physiology are not well understood. The majority of studies rely on field observations, the difficulties of which likely prompted the appearance of controlled laboratory experiments and theoretical studies [21, 31–34].

Considering the range of time spent in burrows across species, and the observed low levels of oxygen saturation in burrow water [27, 35], we tested whether crayfish respiration can quickly deplete burrow oxygen levels and whether survival of these anoxic conditions differs among primary, secondary, and tertiary burrowers. We conducted *in-vivo* and *in-silico* experiments focusing on (i) in-burrow oxygen dynamics during day/night activity cycles, and (ii) tolerance to anoxic conditions of crayfish species from multiple continents that vary in burrowing behaviors. Our data, together with further experimental and molecular investigations, may provide valuable insight into the emerging field of eco-evolutionary feedbacks [36]. We use crayfish as an example of an organism that modifies its environment (i.e., burrow construction) with evolutionary processes potentially affected by the modified environment.

## Material and methods

### Modelling *in-burrow* dissolved oxygen dynamics

#### Simulating dissolved oxygen dynamics in burrow with constant occupancy

**Fig 1:**
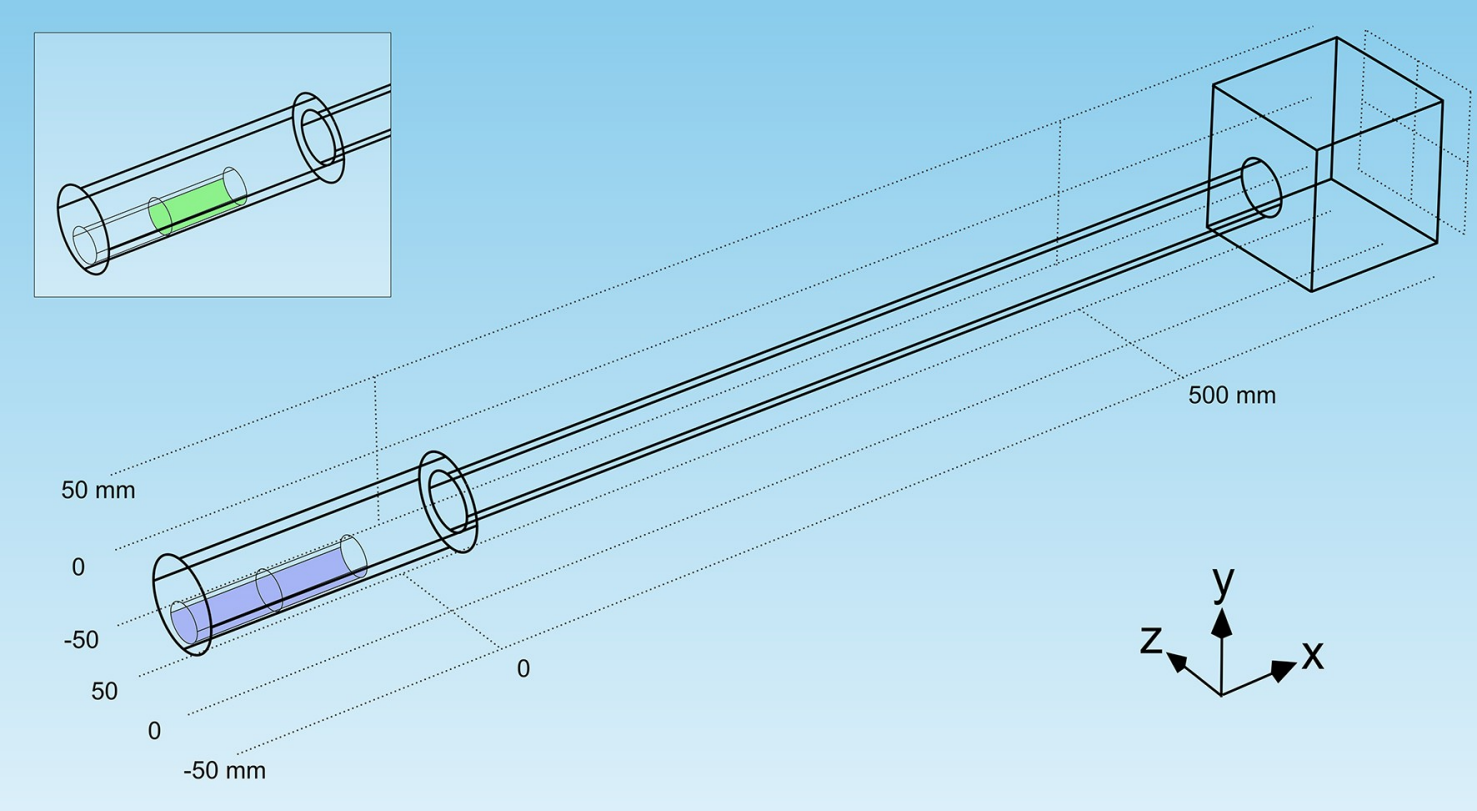
Schematic representation of the geometry of virtual model of crayfish burrow (the walls of the tube were considered impenetrable for oxygen) and a flowing system (the cubic box in which the water is considered flowing, with velocity 0.1 m/s perpendicular to the direction of the burrow). The crayfish is represented by a cylinder (detailed in the image in the left-upper corner), the purple zone represents the moving area of gills and pleopods (imposing a water current of 0,0001 m/s), and the green area represents the consumption zone (the gills).

We developed a mathematical model for oxygen consumption of a virtual crayfish in a virtual burrow. A virtual crayfish with a total length (TL) of 110 mm, 24 mm mean diameter (⌀) and 48 g wet weight (WW) was placed in a virtual cylindrical burrow 180 mm long and 38 mm diameter (⌀), connected by a cylindrical tube (600, 400 or 200 mm long, 30 mm ⌀) to a cubic-shaped external tank (ET) representing a flooded, lateral burrow in the bank of a river or pond (Fig 1). The virtual crayfish was placed with the head oriented towards the exit of the burrow. We placed the consumption area (i.e., the gills) on the ventral side of the proximal half of the virtual crayfish. The local convection currents generated by scaphognathites to maintain oxygen circulation were simulated by imposing a local restricted velocity of 0.0001 m/s [37, 38] on the ventral side of the crayfish where water is drawn into the lateral gill chambers via openings at the base of the legs. The dissolved oxygen (DO) uptake rate (DOUR) was simulated by considering a mass flux type boundary condition (i.e., mass of DO consumed per unit time and unit surface area) on the area of the active surface through which oxygen is consumed.

The relationship between crayfish DOUR and declining DO concentrations was obtained from *in-vivo* empirical data from *Pontastacus leptodactylus*. Methodology was the same as that described in the “Survival of crayfish to self-induced anoxia” section within this manuscript, except that each experimental tank contained only a single, adult crayfish instead of multiple individuals. Trials were run for three males and three females ranging from 31.26 to 47.66 g in males, and 21.39 to 40.01 g in females. We measured DO every 30 minutes as it decreased from 100% saturation to anoxia (0.00 mg O_2_/L). The DOUR of each crayfish was calculated every 30 minutes using the formula:

$$DOUR_{i,i+1}=\frac{(DO_{i}-DO_{i+1})V_{W}}{M\Delta t_{i,i+1}}$$ (1)

where *DOUR*_*i*,*t*+1_ is the oxygen uptake rate per time unit between measurements *i and i+1*, *DO*_*i*_ is DO concentration (mg/L) at measurement *i*, *V*_*w*_ is the volume of water (l) in the respirometer tank, Δ*t*_*i*,*t*+1_ is the time interval (seconds) between measurements *i and i+*1, and *M* is the total wet weight (g) of the crayfish used in experiment. To determine whether background changes in dissolved oxygen were likely to be significant, four control trials were run within the same experimental set-up, but without any crayfish in the Pârvulescu lab (Romania). To initiate control trials, a single crayfish was allowed to draw the DO down in each of four tanks to 8.42, 4.17, 1.22, or 0.06 mg O_2_/L, at which time the crayfish was removed from each tank and the DO was monitored for ten subsequent hours. Dissolved oxygen concentration was then plotted against time (h) and fitted with a linear regression (SigmaPlot 15.0, Systat Software, Inc., San Jose California USA, www.systatsoftware.com).

Within the modeling simulation, the initial DO levels were set at 8.5 mg/L in the ET; we simulated natural flow currents in the ET at a velocity field of 0.1 m/s, 100 mm away from the entrance of the tube in the ET. The oxygen transport inside the virtual burrow by convection and diffusion is described by the equation:

$$\frac{\partial DO}{\partial t}+\vec{v}\cdot \nabla DO=D\Delta DO$$ (2)

where DO is the dissolved oxygen value, t is time, $\vec{v}$ is the velocity field, and D is the diffusion coefficient of oxygen in water.

The walls of the burrow were considered impermeable to oxygen based on literature remarks that usually secondary burrows are made in low coarse and cohesive soil riverbanks [29]. The flow velocity was calculated by numerically solving the classical Navier-Stokes equations for incompressible fluids:

$$\rho (\frac{\partial \vec{v}}{\partial t}+(\vec{v}\cdot \nabla )\cdot \vec{v})=-\nabla p+\mu \Delta \vec{v}$$ (3A)

$$\nabla \cdot \vec{v}=0$$ (3B)

where *ρ* is mass density, *p* is pressure, and *μ* is the dynamic viscosity of water.

The system of Eqs (3A)–(3B) was solved in the previously outlined geometry, together with a non-slip condition imposed on the burrow walls, and a prescribed velocity field of the water at the burrow entrance in the ET. The values of the material parameters used in simulations correspond to the specific values for water at 20°C, density *ρ*_*w*_ = 1000 kg/m^3^, dynamic viscosity *μ*_*w*_ = 0.001 Pa∙s, diffusion coefficient of oxygen in water *D*_*w*_ = 2∙10^−9^ m^2^/s. The time-dependent partial differential equations that describe the mathematical model were solved with the corresponding boundary conditions (Dickinson, Ekström, & Fontes, 2014) by using the Finite Element Analysis software COMSOL Multiphysics 4.3 (COMSOL, Inc., Burlington MA, USA, www.comsol.com).

### Constant occupancy model verification

To verify model predictions, we monitored DO in an artificial burrow inhabited by a live crayfish. In each trial, we placed a single *P*. *leptodactylus* adult male crayfish for 24 h in a cylinder-shaped plastic shelter, hereafter named crayfish chamber (180 mm long, 50 mm ⌀), connected by a 38 mm ⌀ cylindrical plastic tube of 200, 400 or 600 mm length to a 60 L tank filled with water at ≥ 8.5 mg/L DO and temperature between 19–23°C. We prevented the crayfish from escaping by placing an obstacle made of thin wire threads between the tube and crayfish chamber, with no influence on the flow of water or DO variations. The oxygen sensor was placed in the middle of the crayfish chamber, 15 mm from the roof, with automated recording at 30-minute intervals. To mimic diffusion and convection caused by natural flow in a lotic environment, water velocity of 0.1 m/s was produced in the water tank using a submersible pump.

### Simulating DO dynamics in a burrow with cyclical occupancy

Because crayfish may periodically leave and return to their burrows following a diel pattern [31], we simulated burrow DO dynamics under simplified conditions of 12 h occupancy (i.e., day) followed by 12 h of absence (i.e., night), hereafter 12-hour cycles. The model assumed a 600 mm TL, 60 mm ⌀ cylindrical burrow, with the crayfish located at the end of the burrow at the beginning of the simulation. During the 12 h of occupancy, the virtual crayfish was allowed to consume the oxygen from its surroundings according to the previously determined DOUR–DO dependence, followed by 12 h when the crayfish was absent, a period of time when oxygen is freely redistributable in the burrow. When the crayfish “returned” to the burrow, we assumed its location was at the most distant point from the entrance, where DO was in the lower range of normoxia (DO = 6 mg/L).

### Simulating DO dynamics in a non-flooded occupied burrow

To investigate the oxygen dynamics of a burrow with little to no groundwater where crayfish engaged primarily in air-breathing, without a chimney to provide additional ventilation [39, 40], we simulated a crayfish located in a burrow filled with air. In this case, the Eq (3B) is replaced by the mass conservation equation for compressible media (air):

$$\frac{\partial \rho }{\partial t}+\nabla \cdot (\rho \vec{v})=0$$ (2B)

and the values of the material parameters used in simulations correspond to the specific values for air at a pressure of 1 atmosphere and temperature of 20°C: volume density *ρ*_*air*_ = 1.2 kg/m^3^, dynamic viscosity *μ*_*air*_ = 1.85∙10^−5^ Pa∙s, diffusion coefficient of oxygen in air *D*_*air*_ = 1.76∙10^−5^ m^2^/s. We used the same equation of DOUR versus ambient oxygen concentration as from experiments on crayfish in submerged conditions, since oxygen uptake rates have been found to be similar between air and water [41, 42].

### Survival of crayfish to self-induced anoxia

#### *In-vivo* laboratory observations

To document interspecific variation in survival of crayfish to self-induced anoxia, we conducted experiments with 14 species of crayfish placed into three categories based on their position along a burrowing gradient: strong (primary and primary/secondary), moderate (secondary and secondary/tertiary) and weak (tertiary): strong burrowers (*Cambarus striatus*, *Lacunicambarus dalyae*, *Parastacus brasiliensis*), moderate burrowers (*Astacus astacus*, *Pontastacus leptodactylus*, *Faxonius limosus*, *Procambarus clarkii*, *Austropotamobius bihariensis*, *A*. *torrentium*, *Cambaroides japonicus*) and tertiary burrowers (*Faxonius etnieri*, *Pacifastacus leniusculus*, *Cherax quadricarinatus*, *Procambarus vioscai*). Data associated with crayfish (species abbreviations, wet weight) and number of involved individuals and trials can be found in the table and figure files. This selection also reflects the global distribution of crayfish taxa: four European, seven North American, one Oceanic, one Asian, and one South American species. We also tested *Procambarus virginalis*, a parthenogenetic species with a very short evolutionary timescale linked to the aquarium trade [43, 44]. With the exception of *P*. *clarkii*, for which specimens collected from both invaded (European) and native (North American; Alabama) continents were investigated, all other specimens were collected from one location and one population. The number of specimens subjected to experimentation varied depending on their availability for capture in the wild. All experiments were performed on uninjured, adult intermolt crayfish, acclimated for at least one week in laboratory conditions. Food was withheld for 12–24 h prior to experimentation to minimize any potential effects of feeding and digestion.

We measured DO and temperature (T), in a simple oxygen depletion chamber containing dechlorinated, ambient-temperature (between 19–23°C) water, fitted with a submersible pump for homogenization. Because access to experimental systems varied greatly amongst labs, we designed a simple, low-cost system that could be used by all labs. Each laboratory used a small glass aquarium that contained water and a ≥ 4 cm layer of vegetable oil on the surface that prevented oxygen from diffusing across the air-water interface. This design also allowed us to periodically test for mortality via probing crayfish with a rod inserted through the oiled surface. A small, submersible pump gently circulated water within the aquarium to prevent heterogeneity in dissolved oxygen concentrations. We used DO electrodes connected to an oxygen meter to record and store data at 30 minutes intervals between successive measurements. Each DO meter was capable of measuring DO to a precision of 0.01 mg/L and was calibrated before each run according to the manufacturer recommendations. Each experimental run was conducted until either all crayfish were dead, or some crayfish had survived ≥ 9 h under anoxic conditions, whichever came first. Crayfish mortality was assessed by visually inspecting the movements of the body and appendages; specimens were considered dead if their scaphognathites and/or appendages remained inert for more than ten minutes after probing.

For each species, we typically placed multiple crayfish in an aquarium, adapted the volume of water according to specimens’ number and size, and conducted 1–2 runs per species with different specimens used in each run.

To quantify hypoxia tolerance, we calculated lethal concentration (LC) during oxygen depletion as follows: LC50 = the DO concentration at which 50% of crayfish died. Because many crayfish were still alive after DO declined to 0.00 mg/L, we also calculated percent of crayfish surviving to anoxia (0 mg O_2_/L), percent of crayfish surviving ≥ 5 h in anoxic conditions, and percent of crayfish surviving ≥ 9 h in anoxic conditions. Only data for those trials where temperature remained within the 19–23°C and time to reach anoxia fell between 13–27 h were analyzed for survival and presented in the main manuscript. Data for the two remaining species are shown in Table 1B.

**Table 1:** Basic statistics for (A) species kept in the study and (B) species with runs removed from analysis due to methodological issues as indicated by asterisks: lethal concentration (LC) caused by the experimental depletion of oxygen; values expressed in mg/L DO. “Cat.” = burrowing category as defined in Methods. Time to reach anoxia = h taken to reach anoxia for each experiment. LC_50_ = 50% of crayfish died. Percent of crayfish surviving anoxia shows percent surviving to anoxia, percent surviving ≥ 5 h. exposure to anoxia, and percent surviving ≥ 9 h. exposure to anoxia. -- = experiments that were not run long enough to calculate survival.

Species	Cat.	No. of Runs	Time to reach anoxia (h)	No. ind.	Temp. range (°C)	LC_50_ (mg/L)	% of crayfish survival		
							To anoxia	≥ 5 h anoxia	≥ 9 h anoxia
**(A)**									
*Lacunicambarus dalyae*	strong	2	15, 16	11	19.9–22.7	N.A.	100	91	91
*Cambarus striatus*	strong	1	16	4	17.4–23.2	N.A.	100	75	75
*Astacus astacus*	mod.	1	13.5	5	21.6–21.7	N.A.	100	100	80
*Pontastacus leptodactylus*	mod.	2	26.5, 27	20	20.3–22.5	N.A.	100	95	90
*Faxonius limosus*	mod.	2	22.5, 23	20	19.7–21.8	N.A.	95	85	60
*Procambarus clarkii*–EUR	mod.	1	16.5	4	19.7–21.8	N.A.	75	25	25
*Procambarus clarkii*–USA	mod	2	13, 13.5	10	20.5–22.2	N.A.	100	80	30
*Austropotamobius bihariensis*	mod	1	22	11	19.2–20.3	N.A.	64	64	64
*Austropotamobius torrentium*	mod	1	13	4	19.9–20.5	N.A.	100	100	100
*Faxonius etnieri*	weak	1	N.A.	5	19.5–23.4	0.10	0	0	0
*Pacifastacus leniusculus*	weak	2	N.A.	8	19.2–22.0	0.15	0	0	0
*Cherax quadricarinatus*	weak	1	18.5	20	19.3–20.4	0.10	10	10	5
*Procambarus vioscai*	weak	1	22	8	19.2–22.6	0.06	50	0	0
*Procambarus virginalis*	not rated	1	15.5	6	19.0–23.0	0.01	50	50	--
**(B)**									
*Parastacus brasiliensis*	strong	1	25	4	23–25*	0.31	0	0	0
		1	45*	4	22–24*	0.27	0	0	0
*Cambaroides japonicus*	mod.	1	20	11	18–19*	N.A.	63	--*	--*
		1	22.5	11	18–19*	N.A.	54	--*	--*

### Data analyses

The DO (oxygen consumption data) collected near the crayfish during the *in-vivo* experiment, which included varying burrow lengths, were compared to the *in-silico* model predictions for the same experimental conditions. In this respect, we applied paired Wilcoxon signed rank test [45] for each of the three groups according to the lengths of burrow (200, 400 and 600 mm).

To test whether strong and moderate burrowers (combined for analyses) are able to survive in anoxic conditions longer than weak-burrowers, we used the unpaired two-sample Wilcoxon test [45, 46] for each of the three data groups of crayfish survival presented in Table 1. We also used an unpaired two-sample Wilcoxon test to assess whether the time to reach anoxia differed between burrowing groups.

For data management, exploratory and statistical analyses, we used R 4.0.3 software [47] using the *wilcox*.*test* function.

### Ethics

The crayfish used in our experiments were treated as humanely as possible within the limitations of the method employed. For the protected species in Europe (*A*. *torrentium*, *A*. *bihariensis* and *A*. *astacus*), specific approvals were obtained before the onset of the project from the Romanian Academy (permit number: 2257/CJ/21.12.2009) and Ministry of Environment in Romania (permits number: 423/19.02.2010 and 1404/18.02.2010), under supervision of the Iron Gate Nature Park Administration (permit number: 136/20.01.2010). Samples of the threatened *C*. *japonicus* were collected from out of National Park, in this case do not require permitting of Japanese Government staff. The species *P*. *leptodactylus*, *P*. *leniusculus*, *C*. *quadricarinatus*, *C*. *striatus*, *L*. *dalyae*, *P*. *brasiliensis*, *P*. *virginalis*, *P*. *clarkii* and *P*. *vioscai* are not threatened or protected and does not required permission in any of the countries.

## Results

### Modelling in-burrow dissolved oxygen dynamics

#### Simulating DO dynamics in burrow with constant occupancy

**Fig 2:**
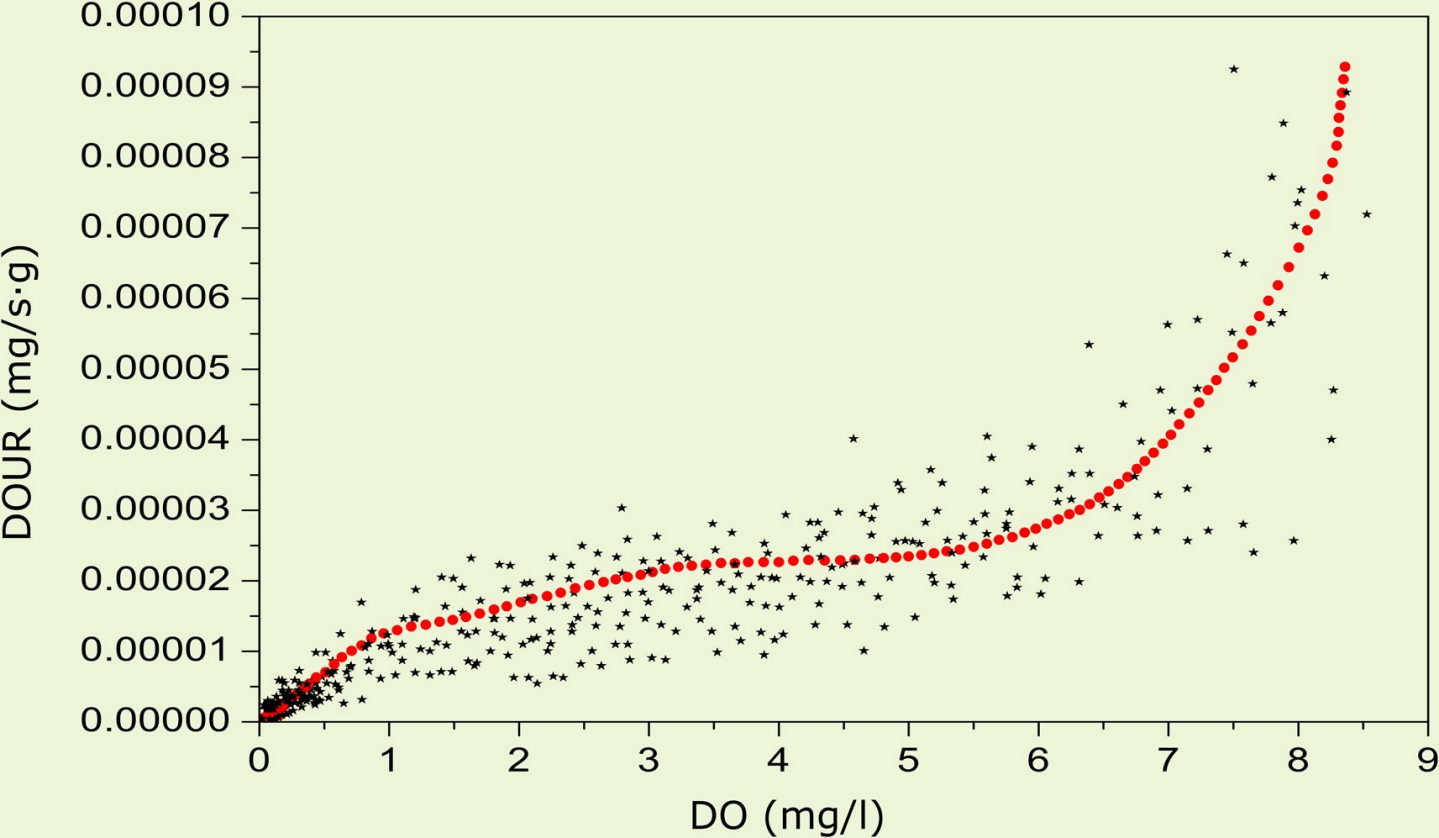
The dissolved oxygen behavior graphs based on merged data for *Pontastacus leptodactylus*. Black stars indicate experimental measurements and red dots represent predicted values based on mathematical model. The best fit (r^2^ = 0.91) was obtained for polynomial function of order 9.

Data used to estimate oxygen consumption rates of individual crayfish (*P*. *leptodactylus*) under conditions of declining DO for development of the burrow model are shown in Fig 2. Control runs (no crayfish in tank) revealed a slight but significant (*p* < 0.05) linear increase, rather than decrease, in oxygen over ten h, suggesting some diffusion of oxygen through the oil layer. However, the rate of diffusion was negligible, ranging from 0.0008 mg O_2_/L/h under normoxic conditions (i.e., initial DO of 8.42 mg O_2_/h) to 0.0037 mg O_2_/L/h under near anoxic conditions (i.e., initial DO of 0.06 mg O_2_/L/h).

**Fig 3:**
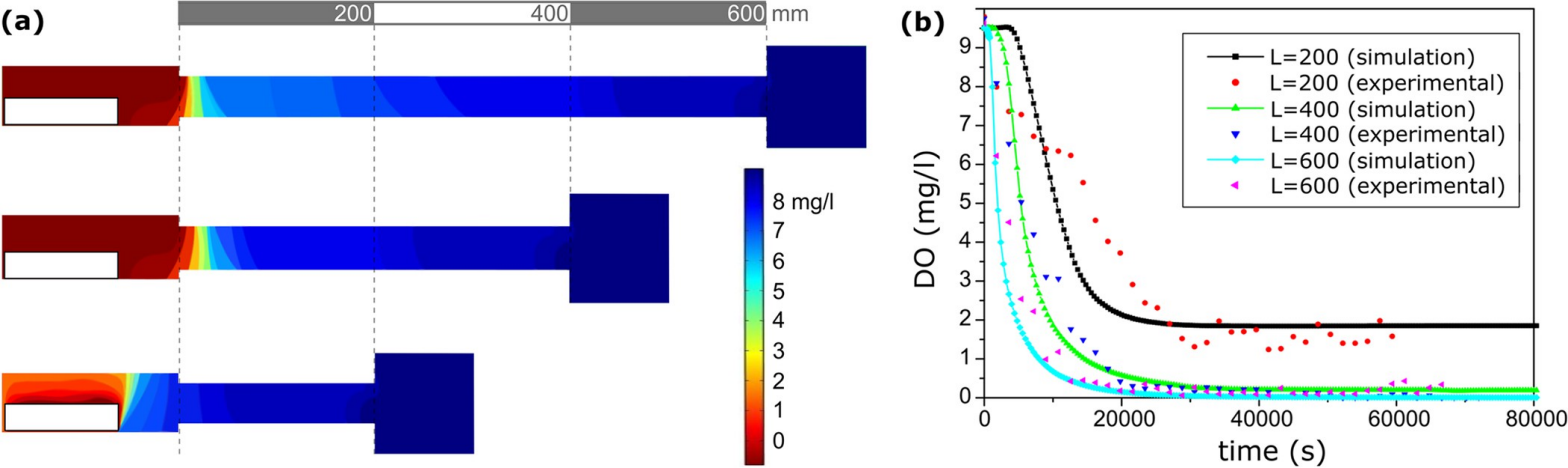
The modeled DO distribution inside simulated crayfish burrows of three lengths is shown in figures (a). Simulated and experimental oxygen consumption behavior for *in-silico* experiments performed for three different burrow lengths, in mm (b).

Simulations using the completed *in-silico* model showed that after 30,000 s (about 8 h), DO declined to near zero at the location of the virtual crayfish in simulated burrows with 600- and 400- mm length connecting tubes (Fig 3A). However, in the shorter 200 mm burrows, DO values near the simulated crayfish did not reach anoxia. Rather, the oxygen delivered through convection from external water flow allowed DO to remain > 6 mg/L in some portions of the burrow proximal to the crayfish (Fig 3A). We found no significant differences (*p* = 0.613, 0.981 and 0.399 respectively) in DO between *in-vivo* experiments, in which a live crayfish was placed in an artificial burrow, and model predictions for each of the three lengths (Fig 3B).

#### Simulating DO dynamics in burrow with cyclical occupancy

**Fig 4:**
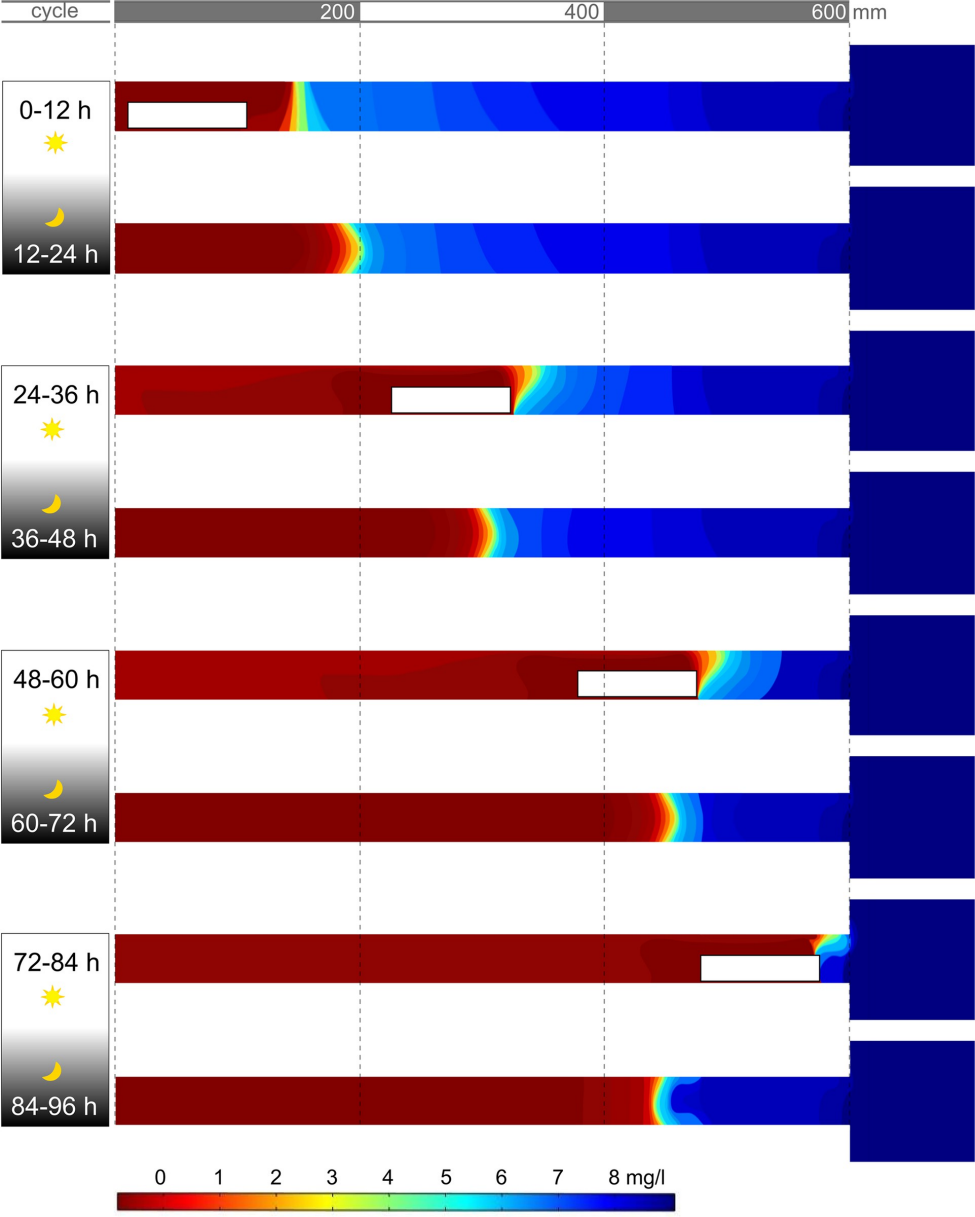
Calculated dissolved oxygen distribution inside the burrow after successive 12-hour day and night cycles. The model considers that the crayfish (the white rectangular) occupies the shelter and consumes oxygen during the day. During the night, when the modeled crayfish leaves the shelter, supplementary oxygen is provided to the burrow by diffusion from the outside water.

Our simulations on DO consumption in a virtual burrow in multiple 12 h cycles (Fig 4) show that an immobile crayfish can consume almost all oxygen in the first 12 h when occupying burrows longer than 400 mm. In our model, the oxygen in the burrow did not return to initial, pre-inhabitancy levels during the next 12 h with no crayfish, indicating that the external water-flow-induced convection is not sufficient to completely replenish DO in the burrows with 600- and 400- mm length connecting tubes. Of note, after four such in-and-out cycles, these burrows were basically depleted of oxygen. Consistent with the DO modelling (and *in-vivo* experimental measurements), the convection effect was relevant only for burrows with a 200 mm connecting tube (or shorter), suggesting that only crayfish close to the ET would benefit from continuous oxygen supply from outside.

#### Simulating DO dynamics in a non-flooded occupied burrow

The *in-silico* simulations for an air-filled burrow, specifically relevant to primary burrowers, showed only a thin line of oxygen depletion around the consumption area of the crayfish, with the rest of the burrow volume remaining oxygen-saturated. This was primarily due to the rate of diffusion in air being much higher than in water.

### Survival of crayfish in self-induced anoxia

#### Hypoxia and anoxia tolerance

Two species (*P*. *brasiliensis* and *C*. *japonicus*) were removed from analysis due to methodological issues related to temperature, time to reach anoxia, and time exposed to anoxia falling outside of designated limits. Data for these species are provided in the table and figure files. Within the remaining fourteen species, burrowing groups did not differ in their time to reach anoxia (*p* = 0.84). However, the percent of individuals surviving to anoxia, surviving ≥ 5 h anoxia, and surviving ≥ 9 h anoxia was significantly lower for the weak burrowers group than for the combined strong and moderate burrower group (*p* < 0.01 for all three survival endpoints). All weak burrowing species exhibited LC50 values > 0.00 mg/L, and two of the four species exhibiting LC100 > 0.00 mg/L. Only a single, weak burrowing species exhibited any tolerance of anoxia with 5% surviving ≥ 5 h and 2% surviving ≥ 9 h (Table 1A). Conversely, all strong and moderate burrowing species showed some tolerance of anoxia, exhibiting 64–100% survival to anoxia. Once anoxia was reached, 25–100% of individuals from strong and moderate burrowing species survived ≥ 5 h, and 25–91% survived ≥ 9 h (Table 1A). Only 25% of invasive *P*. *clarkii* from the European site survived ≥ 5 and ≥ 9 h, while 80% of *P*. *clarkii* from the North American site survived ≥ 5 h but only 30% survived ≥ 9 h. Fifty percent of the aquarium species, *P*. *virginalis*, survived to anoxia and fifty percent survived ≥ 5 h of anoxia (Table 1A). In all trials, DO consistently remained at 0.00 mg O_2_/L for the subsequent > 9 h monitoring period after reaching anoxia.

## Discussion

### Ecological implications

Generally, crayfish behave differently under varying environmental conditions [48]. The ability of some species to survive for considerable period of time in poor oxygen conditions would be a distinct advantage in harsh environments where drought and extreme temperature may be typical [49]. Multiple observational field results showed that the water in inhabited crayfish burrows is essentially hypoxic and acidic (with pH reaching values as low as 3.8 in the galleries of *Parastacoides tasmanicus*), a pattern influenced by limited air–water exchanges and crayfish respiration [50, 51]. To explain their survival in severe hypoxic conditions, it was suggested that crayfish are actively positioning themselves at air–water interfaces, or aerating burrows via passive ventilation structures, thus procuring the necessary oxygen directly from air [27, 39]. Here, we show that crayfish respiration in fully submerged burrows would be sufficient to rapidly (ca. 8 h) reduce DO in the surrounding burrow water even when initial DO concentrations are high (i.e., 8.5 mg/L), shown with high concordance between our *in-silico* and *in-vivo* experiments. Our experiments show that severe hypoxia and anoxia are inherent events in submerged crayfish burrows, primarily dependent on the burrow length from the terminal end to the source of flowing, oxygenated water. Crayfish adaptation to hypoxia involves behavioral and physiological responses such as changes in scaphognathite beating rates and cardiac rhythm [52], and osmotic [22] and biochemical changes of haemocyanin-O_2_ affinity mechanisms (anaerobic metabolic switch with quick lactate build-up in the hemolymph) [53, 54], presumably reflecting ecological and evolutionary aspects. Some crayfish escape hypoxia by reaching air–water interfaces [54]; however, this behavior would not be possible in fully flooded, lateral burrows modeled in this study. Some crayfish species do not show preference for oxygenated waters when offered the choice, indicating a good tolerance for hypoxia [55, 56].

Primary burrowing crayfish spend most of their life in elaborate burrows disconnected from running, oxygenated waters, from which they emerge for mating and food foraging [17, 57]. Even though these burrows contain an air/water interface, conditions inside the flooded portions of these burrows are harsh: the dissolved oxygen levels might reach as low as 0.7 mg/L and a pH below 4.5 [51]. Tertiary burrowing crayfish spend the vast majority of time in surface waters, infrequently retreating into their simple, shallow burrows for temporary avoidance of predators or desiccation, while secondary burrowers exhibit intermediate burrowing behavior. Given these burrowing behaviors and conditions, we would have expected the strong burrowers to perform the best in our hypoxia/anoxia experiments, followed by the moderate and then by the weak burrowers. Insufficient numbers of primary burrowing species and variation in experimental conditions precluded separate, quantitative comparisons between strong burrowers and the other two groups. However, results clearly showed that both strong burrowing species tested, as well as the moderate burrowers had high tolerances for anoxic conditions. Strong and moderate burrowing species were highly tolerant of hypoxia and survived many h of complete anoxia (i.e., 0.00 mg O_2_/L), often exhibiting ≥ 60% survival of anoxia for 9 h or more. In contrast, weak burrowers were highly sensitive to hypoxia with only one species (*C*. *quadricarinatus*) showing limited survival of anoxia. These findings suggest that the micro-ecology of aerated and flooded burrows of primary and secondary burrowers have provided the ideal conditions to consistently conserve the physiological and metabolic mechanisms of hypoxia tolerance (see sections below), as opposed to the infrequent, shallow temporary shelters of tertiary burrowers. It is worth noting that the aquarium species *P*. *virginalis* behaved somewhere between moderate and weak burrowing groups.

Significant challenges when conducting experiments on multiple continents, across multiple labs with differing research resources included ensuring that experimental conditions remained similar among trials and predicting the biomass per volume required to bring DO to anoxia within a specific time range. More species need to be examined under consistent conditions to better test for consistent differences between burrowing categories. However, results clearly demonstrate a high tolerance of anoxic conditions in strong and moderate burrowers–a tolerance that appears to be much reduced in tertiary burrowers.

Burrowing behavior of crayfish may change in invaded habitats, which raises interesting questions as to whether anoxia tolerance also changes. For example, the Signal Crayfish (*P*. *leniusculus*) is considered a tertiary burrower in its native, North American range but may behave more like a secondary burrower within invasive European ranges (Guan, 1994). We found little to no evidence for changes in anoxia tolerance in invasive populations. In our study, *P*. *leniusculus* collected from an invasive European population in Czech Republic showed no tolerance of anoxia. Red Swamp Crayfish (*P*. *clarkii*) showed moderate tolerance of anoxia, with 25–30% surviving ≥ 9 h of anoxia regardless of whether they were collected near the edge of their native range (i.e., Alabama, United States), or from a distant, invasive population (Hungary). Additional studies are needed to determine the frequency with which invasive populations change their burrowing behaviors and the degree to which anoxia tolerance may differ between native and invasive populations.

### Physiological implications

Although some crayfish species may gain protection from adverse conditions (i.e., thermal stress, desiccation, predation) by creating modified habitat in the form of burrows, they also face a trade-off in that the burrow water is likely to quickly go hypoxic or anoxic. Thus, it is to be expected that a wide range of crayfish species have evolved physiological adaptations to withstand hypoxia or even anoxia for many h at a time. This is supported by results of this study wherein crayfish taxa from multiple continents, families, and burrowing groups exhibited LC50’s < 0.5 mg O_2_/L and were capable of surviving many h of complete anoxia.

Adaptation to hypoxic and anoxic regimens is accompanied by a significant divergence between the hyperglycemic and lactataemic hemolymph responses in early and late anoxia [58]. This indicates that, at least up to a certain level of anoxia, the ability to respond to the lack of oxygen depends on the individuals’ ability to mobilize energetic substrates (from hepatopancreas and muscles) [59]. Hemolymph lactate accumulation, a specific response of crustaceans exposed to hypoxic conditions, is significantly increased below the critical point and represents the sign of metabolic switch to anaerobiosis as the animals start to oxyconform [23, 54, 60, 61]. The continuous accumulation of lactate is surprising given that the affinity of hemocyanin for oxygen decreases in parallel with lactate [62], which would improve the oxygen release in peripheral tissues. The hemolymph hyperglycemic response to hypoxia has been previously documented in crustaceans [60, 63–65]. The hyperglycemic response to anoxia with an abrupt drop after 3 h of anoxia (preliminary data, not included in this study) is a reminder of the hypoxia experiments on the freshwater crab, *Eriocheir sinensis* [66], *Parastacus defossus* [60], and the intertidal crab *Chasmagnathus granulata* [67]. A possible scenario explaining the anoxia resilience would involve activation of gluconeogenetic mechanisms [68, 69] and rapid mobilization of muscle and hepatopancreas glycogen to glucose, with its subsequent anaerobic use to lactate, and the usage of arginine phosphate (a well-known ATP buffer for ATP during hypoxia) [60]. During anoxia, glycogen mobilization is gradually exhausted and hemolymph glucose level drops, possibly due to muscle re-synthesis [70], while the still accumulating hemolymph lactate is being used as for ATP production by an anoxia-adapted [24] lactate dehydrogenase (LDH).

Similar to changes observed in other organisms, crustacean adaptation to hypoxia involves time-dependent, tissue-specific changes in HIF-1α and HIF-1β expression levels [71, 72], and dysregulation of expression in hypoxia associated microRNAs (miR-210, let-7, miR-143, and miR-101) [73], with the possible establishment of a HIF-miR feedback loop. It has been shown that HIF has a dual regulatory role upon glycolysis, with upregulation of phosphofructokinase (PFK) in short-term hypoxic conditions and upregulation of fructose bisphosphatase (FBP) in long term hypoxia [74]. Of note, HIF-1 silencing in shrimps subjected to hypoxia leads to reduced LDH activity and lactate accumulation, underlying the role of HIF-1 in crustacean adaptation to hypoxia [74].

### Evolutionary implications

Of the over 540 species of crayfish [13], there are well documented inter- and intra-specific variations in burrowing behavior with some species having primary, secondary or even tertiary burrower populations [16, 75]. Our work indicates that in addition to morphological and genetic characteristics [76–78], another layer of complexity could be taken into consideration for the classification of freshwater crayfish: the ability to withstand severe hypoxia/anoxia. It is worth noting that the separation into the three classical clades (Astacidae, Parastacidae, and Cambaridae) does not parallel the freshwater crayfish’s ability to withstand severe hypoxia/anoxia. The existence of primary burrowers among the American and Australian continents, and the lack of primary burrowers in European species, suggests that the different burrowing behaviors developed after the crayfish Jurassic colonization of freshwater, in parallel with the establishment of the different crayfish families under different evolutionary ecological pressure [79].

The origin of the mechanisms behind the abilities of crayfish to cope with anoxia is perhaps reflective of evolutionary ecological drivers. Having lobster ancestors, the crayfish transition to freshwater habitats occurred hundreds of millions of years ago [7, 80, 81]. Most likely, the crayfish legacy is strongly related to their ancestors; nonetheless, what is ecologically preserved from this heritage is still debatable. Lobsters obtain energy anaerobically to survive migration across deep ocean waters with low oxygenation [82–84]. This situation is rare in freshwater habitats, except in parts of deep, productive lakes which are often avoidable during migrations. Yet, these anaerobic mechanisms appear to have been preserved due to crayfish use of burrows–particularly flooded burrows.

Crayfish are susceptible to predation, cannibalism, and desiccation, therefore sheltering in burrows is a common behavior. Burrowing in flooded shelters appears to be a very old behavior based on the existence of fossilized crayfish burrows [85]. Recent adaptations, such as those generated by cavernicol or hyporheic life, did not significantly affect crustaceans’ genetic heritage, making the secondary adaptations reversible [86, 87]. A central question is whether freshwater crayfish were already adapted to hypoxic conditions due to ancestral characteristics or whether hypoxia tolerance evolved in response to conditions in newly engineered environments. Since the ancestors of crayfish are (monophyletically) the lobsters [80] and they are capable of burrowing but do not behave like primary burrowers, it is possible that the main root of crayfish taxa is the secondary type, from which the primary and tertiary burrowers have split. We therefore propose a new hypothesis from an eco-evolutionary perspective, the main branch being the secondary burrowers from whom emerged the primary (a.k.a., constructors of aerated burrows, or fossorial) and tertiary burrowers (lacking burrowing requirements) due to specific evolutionary challenges as crayfish moved from a marine to a variety of freshwater terrestrial habitats. However, more interdisciplinary ecological and molecular studies are needed to solve the complex aspects that drove ecological split(s) among phylogenetically similar crayfish.

In conclusion, we found crayfish taxa that engineer beneficial habitats in the form of burrows can also negatively affect these habitats by driving burrow water to anoxia. A wide range of strong and moderate burrowing taxa from multiple continents appear to have either evolved or retained an ancestral ability to tolerate hypoxic and even anoxic conditions for a considerable amount of time, as compared to weakly burrowing taxa with limited tolerance of hypoxic and/or anoxic conditions. The relative contributions of genotypic and phenotypic adaptations to hypoxia tolerance deserve further study. For example, anoxia is relevant in shaping juveniles’ development in some species, protecting their heart from further hypoxic stress [88]. The evolutionary selection process of hypoxia dwelling gene complexes may be particularly important during early juvenile stages when they share a common burrow with their mother [68, 89]. Further research will be of great use in teasing out the interactions and feedbacks between ambient (i.e., streams) and engineered (i.e., burrows) environmental characteristics and the evolution of environmental tolerance traits across diverse crayfish taxa.
